# Assessment of Non-alcoholic Fatty Liver Disease and Level of Risk of Fibrosis in Diabetic and Non-diabetic Individuals

**DOI:** 10.7759/cureus.76162

**Published:** 2024-12-21

**Authors:** Miah Wahiduzzaman, Noor-E- Ferdous, K. M. Mozibul Haque, A. K. M. Shamsul Kabir, Md. Adib Siddiki, Md. Tanim Hossain, Qazi Ashrafur Rahman, Al Istiak Ur Rahman, A. H. M. Golam Kibria

**Affiliations:** 1 Medicine, Holy Family Red Crescent Medical College and Hospital, Dhaka, BGD; 2 Gynecological Oncology, Bangabandhu Sheikh Mujib Medical University, Dhaka, BGD; 3 Anesthesiology and ICU, Holy Family Red Crescent Medical College and Hospital, Dhaka, BGD; 4 Marketing Pharma-Human, Renata Public Limited Company, Dhaka, BGD; 5 Epidemiology and Biostatistics, Center for Medical Research and Development (CMRD), Dhaka, BGD

**Keywords:** family history of liver disease, fibrosis-4 index, metabolic dysfunction-associated steatotic liver disease (masld), non-alcoholic fatty liver disease (nafld), non-diabetic individuals, type 2 diabetes mellitus (t2dm), ultrasonography

## Abstract

Background and aim

Non-alcoholic fatty liver disease (NAFLD), now known as metabolic dysfunction-associated steatotic liver disease (MASLD), is more common in people with type-2 diabetes mellitus (T2DM) than in people without diabetes mellitus (non-DM). This disease can lead to cirrhosis or hepatic cancer. There is limited data on NAFLD prevalence and the level of risk of fibrosis in Bangladeshi individuals. This study aimed to assess NAFLD prevalence and compare the proportion of NAFLD and the level of risk of fibrosis between T2DM and non-DM Bangladeshi individuals.

Methods

A cross-sectional analytical study was conducted for six months in 2024 in the outpatient section of the Department of Medicine at Holy Family Red Crescent Medical College, Dhaka, Bangladesh. Among the patients seeking outpatient care, a total of 179 male and non-pregnant female participants aged 18 years and older were selected using a purposive sampling technique. Individuals with a history of alcohol use, diagnosed cases of chronic liver diseases, prior use of hepatotoxic drugs, and primary biliary cholangitis were excluded from the study. Detailed demographic characteristics, comorbidities, family history of diabetes and liver disease, physical measurements, and biochemical tests were done. Ultrasonography (USG) of the hepatobiliary system was employed to ascertain the existence of NAFLD. The presence or absence of T2DM was evaluated through prior medical documents, corroborated by laboratory analyses of random blood glucose (RBS) and glycosylated hemoglobin (HbA1c) levels. The Fibrosis-4 (FIB-4) index score was utilized to evaluate the risk of liver fibrosis.

Results

The mean age of the participants was 49.11±12.25 years and 107 (59.8%) of participants were female. Almost two-thirds of the participants were suffering from T2DM. About 17 (9.5%) of the study participants were suffering from NAFLD, which was much higher among T2DM (15 (12.5%)) than non-DM individuals (two (3.3%)). T2DM and family history of liver disease were found to significantly increase the risk of suffering from NAFLD by 5.247 times (95% CI: 1.081-25.468) and 4.202 times (95% CI: 1.249-14.135), respectively. About one (6.7%) of T2DM individuals with NAFLD were at high risk for fibrosis.

Conclusion

Almost one in 10 people had NAFLD, and it was way more common among those with T2DM, who also exhibit a higher risk of hepatic fibrosis. Moreover, T2DM and a family history of liver disease can independently increase the risk of NAFLD.

## Introduction

Non-alcoholic fatty liver disease (NAFLD), also known as metabolic dysfunction-associated steatotic liver disease (MASLD), is characterized by the accumulation of excess fat in more than 5% of liver cells in individuals who consume minimal alcohol (defined as <20 g/d for females or <30 g/d for males) and do not have other secondary causes such as medications, starvation, or monogenic disorders [1]. NAFLD is thought to affect 32% of adults worldwide, with males having a higher incidence (40%) than females (26%). According to hospital-based research, the prevalence of NAFLD has increased, rising from 26% in studies done in 2005 or earlier to 38% in studies done in 2016 [2]. In Western countries, the prevalence of NAFLD ranges from 20% to 30% [3]. With a usual range of 15% to 30%, the prevalence percentage in the Western world is almost mirrored in the Middle East, Japan, and China. The prevalence of NAFLD varies throughout Asia. There is evidence that 16%-32% of urban residents and 9%-16% of rural residents in the Indian subcontinent suffer from NAFLD [3,4]. In the general population of Bangladesh, the prevalence of NAFLD ranges from 4% to 18.4% [5]. Alam et al. did a study that revealed that around one-third of Bangladesh's population suffers from NAFLD [6]. Obesity, diabetes, dyslipidemia, insulin resistance, and metabolic syndrome are recognized as factors related to the onset of NAFLD [7]. According to studies conducted in Bangladesh, midlife adults, married, mostly women from rural areas, being overweight or obese, suffering from diabetes mellitus (DM), having high blood pressure (HTN), having metabolic syndrome, and suffering from dyslipidemia had a higher chance of getting NAFLD compared to others [6,8]. The prevalence of NAFLD among diabetic patients in Bangladesh is 49.8% [5]. According to Rahman et al., the chance of getting NAFLD is 2.21 (with a confidence interval of 1.43 to 3.41 times) times higher in people with diabetes [8].

Simple steatosis, steatohepatitis, fibrosis, and cirrhosis are the numerous phases that NAFLD can progress through, and it may even finally result in hepatocellular carcinoma [7,9]. The progression of fibrosis to cirrhosis and hepatocellular carcinoma can occur over the course of several years. Clinicians must prioritize the early detection of fibrosis to prevent terrifying sequelae. Liver biopsies are the diagnostic gold standard for fibrosis, but they are invasive and not always available, therefore researchers are looking into less invasive alternatives. One of the most common non-invasive risk assessment tools utilized nowadays is the Fibrosis-4 (FIB-4) score. According to Angulo et al., the FIB-4 score performs better than other measures due to its superior predictive power and better diagnosis rate in high-risk patients [10]. In patients with NAFLD, a FIB-4 score of >2.67 is considered indicative of the onset of fibrosis [11].

Global NAFLD and diabetes prevalence is expanding [12,13]. Due to shifting food habits and sedentary lifestyles, there are more cases of diabetes and NAFLD in Bangladesh [14,15]. The prevalence of NAFLD among individuals with type-2 DM (T2DM) shows significant global variation, with rates spanning from 34% to 94% [16]. Research indicates that 30%-40% of patients with NASH exhibit advanced liver fibrosis upon presentation, whereas 10%-15% have developed cirrhosis [17]. T2DM significantly influences the prevalence and severity of NAFLD). The mortality rate in diabetic patients with cirrhosis exceeds twice that of the general population. Individuals diagnosed with both non-alcoholic steatohepatitis (NASH) and T2DM demonstrate a poorer prognosis, marked by increased rates of cirrhosis and mortality [18]. Moreover, NAFLD imposes a significant economic burden on Bangladesh's healthcare system. The financial burden of illness escalates as NAFLD advances to cirrhosis [19].

The existing information regarding NAFLD in Bangladesh is scarce. Considering the increasing occurrence of diabetes and NAFLD in Bangladesh, as well as the related economic consequences, it is crucial to examine the prevalence of NAFLD and the risk of fibrosis within the Bangladeshi population. This study sought to evaluate the prevalence of NAFLD and to analyze the differences in the proportion of NAFLD and the associated risk of fibrosis between individuals with T2DM and those without diabetes in Bangladesh.

## Materials and methods

A cross-sectional analytical study was conducted in the Department of Medicine at Holy Family Red Crescent Medical College, Dhaka, Bangladesh, spanning from January 2024 to June 2024. Patients attending the outpatient section of the Department of Medicine to seek medical care were approached for the study. The study included 179 patients aged 18 years and older, consisting of male and non-pregnant female participants, selected through a purposive sampling technique. Participants with a background of alcohol consumption (≥20 g/d for females or ≥30 g/d for males), diagnosed cases of chronic liver diseases, previous use of hepatotoxic medications, and primary biliary cholangitis were not included in the study. After clarifying the study's objectives, informed written consent was gained from the participants. The patient's medical history was carefully recorded, encompassing demographic information, comorbidities, family history of DM and liver disease, along with physical measurements such as body mass index (BMI), abdominal girth, and blood pressure readings (systolic and diastolic blood pressure). Additionally, biochemical tests including lipid profile, complete blood count, platelet count, aspartate aminotransferase (AST), alanine aminotransferase (ALT), and albumin levels were included. Following an overnight fast, ultrasonography (USG) of the hepatobiliary system and biochemical analyses were performed. The existence or absence of T2DM among participants was determined by prior medical records, corroborated by laboratory assessments of random blood sugar (RBS) and glycosylated hemoglobin (HbA1c), following the recommendations established by the American Diabetes Association [20]. Data were collected by a face-to-face interview using a preformed semi-structured questionnaire (data collection sheet). The formal ethical approval for this study was obtained from the ethical committee of Holy Family Red Crescent Medical College (Memo No.: Holy/Admn./1004/423/2023).

Diagnosis of NAFLD

A radiologist specializing in hepatobiliary system USG was asked to evaluate NAFLD presence or absence. Two of the three established criteria described by Quinn and Gosink helped to identify NAFLD: (1) enhanced hepatic echogenicity in relation to the spleen or kidney; (2) liver vascular obscuration; and (3) intense attenuation of the ultrasonic signal [21].

Assessment of the risk of liver fibrosis

**Figure 1:**
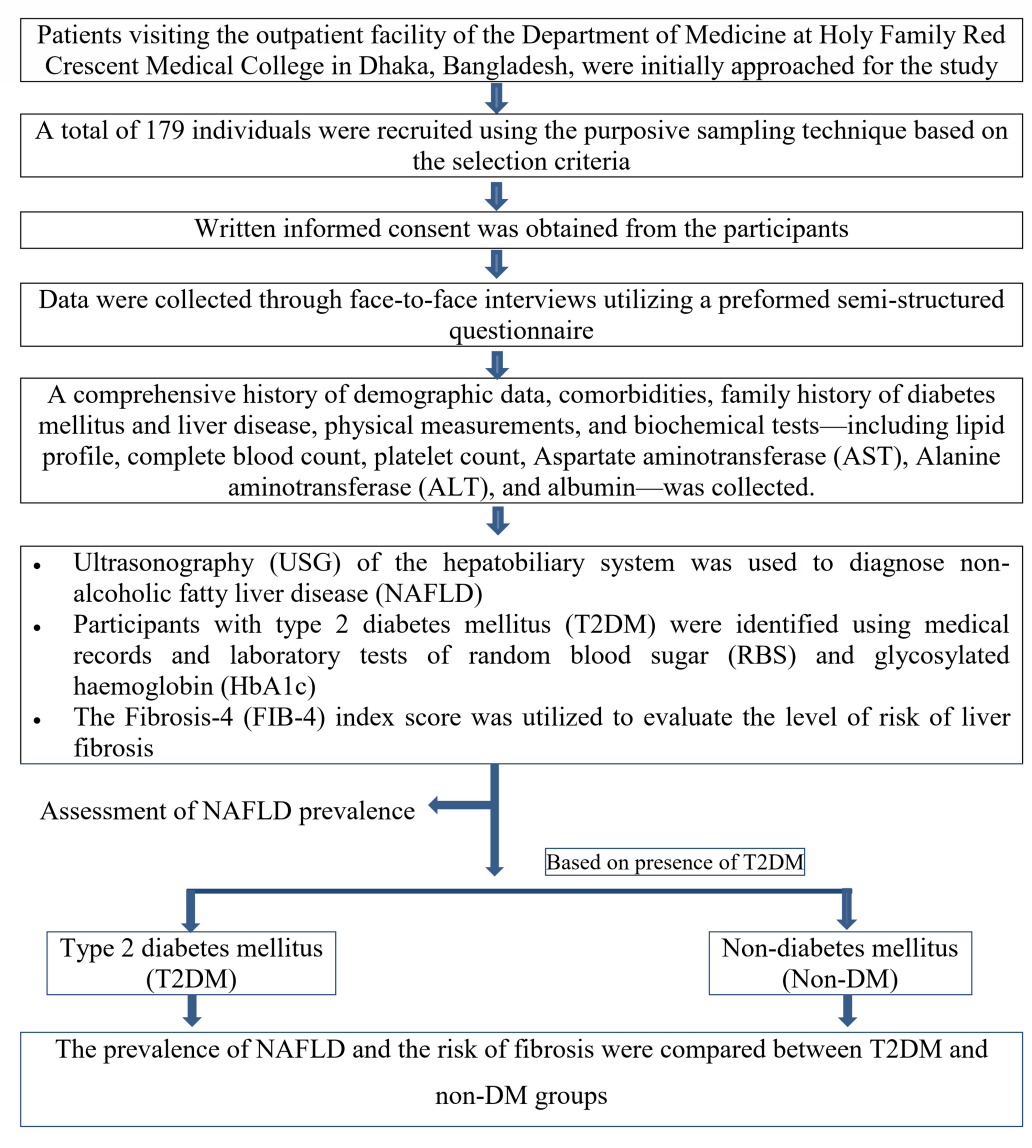
Study flow chart

The FIB-4 index score was employed to assess the risk of liver fibrosis and to estimate the probability of progression to cirrhosis over the following two years. The FIB-4 index score is a calculated metric utilized to evaluate risk levels in individuals with NAFLD. It incorporates age and specific laboratory tests, including AST, ALT, and platelet count. The baseline FIB-4 score was categorized based on the risk of advanced fibrosis into three distinct classifications: low risk (<1.30), indeterminate risk (1.30-2.67), and high risk (>2.67) [22]. Figure 1 presents a flow diagram of the study.

Statistical analysis

The data analysis was performed utilizing the Statistical Package for Social Sciences (SPSS) version 23.0 (IBM Corp., Armonk, NY). Continuous variables were summarized by determining the mean, standard deviation, median, and range. Frequency distributions were employed to summarize category variables. The Shapiro-Wilk test was employed to evaluate the normality of the data distribution. The association between categorical variables and the presence or absence of NAFLD was assessed using the chi-square test or Fisher’s exact test, depending on the suitability of the data. Risk variables for NAFLD, such as T2DM, familial diabetes history, and familial liver disease history, were evaluated by univariate and multivariate logistic regression analyses. The results are presented as odds ratios, along with their corresponding 95% confidence intervals. A p-value less than 0.05 is deemed to signify a statistically significant difference.

## Results

The mean age of the participants was 49.11±12.25 years, and a majority (98 (54.7%)) were from the 41-60 age group. The majority (107 (59.8%)) of participants were female. The majority of the participants were higher secondary school certificate-passed (HSC) (53 (29.6%)) and unemployed (101 (56.4%)). The median monthly income of the participants was 60,000 takas (Table 1).

**Table 1:** Distribution of the participants according to demographic characteristics (n=179) SD - Standard deviation, SSC - Secondary school certificate, HSC - Higher Secondary school certificate Data were presented as Mean ±SD, Median (range), Frequency (%)

Demographic characteristics		Values
Age (years)	18-40	53 (29.6)
	41-60	98 (54.7)
	>60	28 (15.6)
	Mean ±SD	49.11±12.25
	Median (range)	50 (18-77)
Gender	Male	72 (40.2)
	Female	107 (59.8)
Education	Below SSC	39 (21.8)
	SSC	25 (14.0)
	HSC	53 (29.6)
	Honors	52 (29.1)
	Masters and above	10 (5.6)
Occupation	Employed	51 (28.5)
	Unemployed	101 (56.4)
	Businessman	4 (2.2)
	Retired	13 (7.3)
	Others	5 (2.8)
	Students	5 (2.8)
Monthly income (Taka) (n=59)	Mean ±SD	54771.19±19099.63
	Median (range)	60000 (10000-100000)

**Figure 2:**
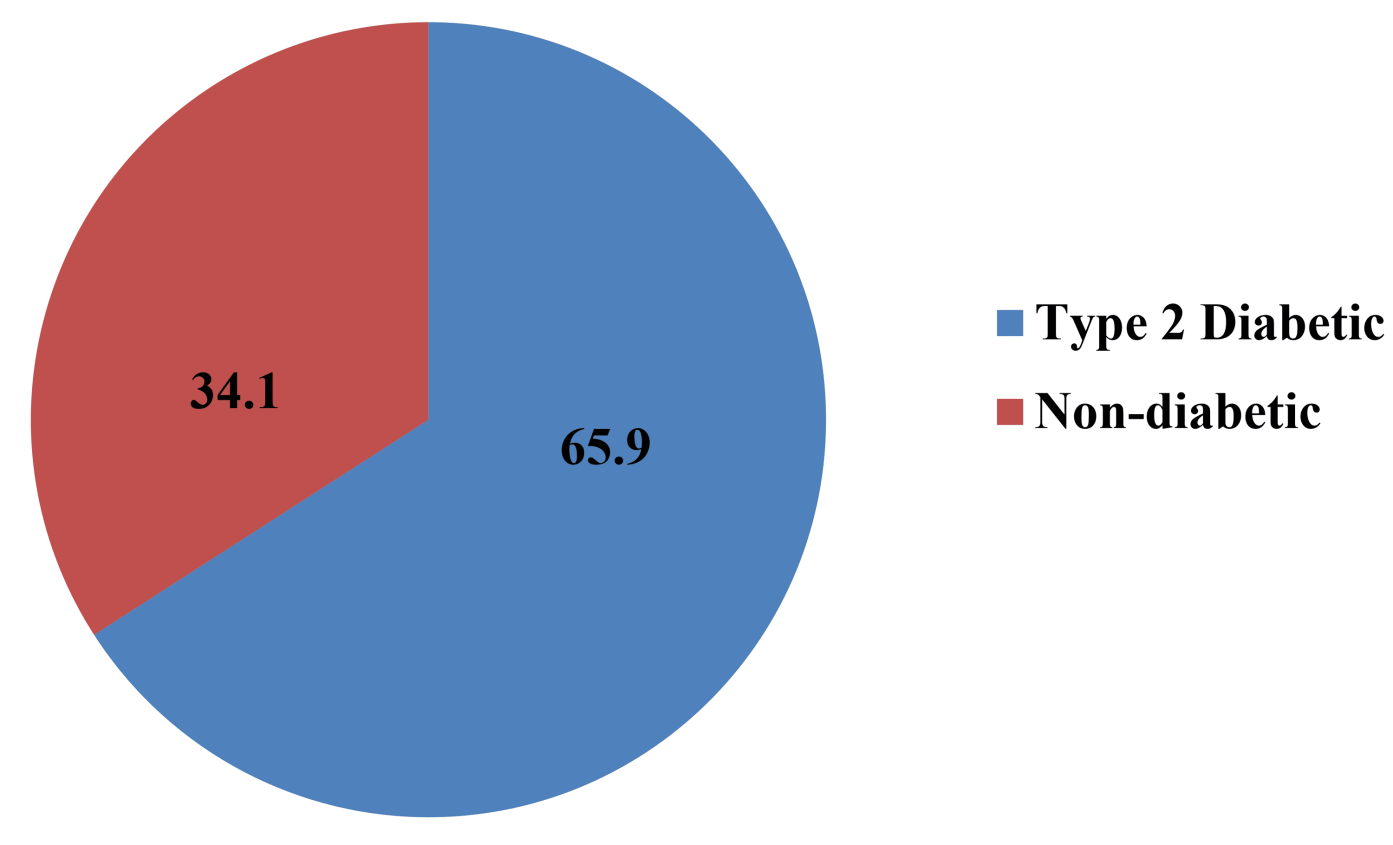
Proportion of type-2 diabetic and non-diabetic patients among study participants

Among the study participants, 118 (65.9%) were suffering from T2DM (Figure 2).

**Figure 3:**
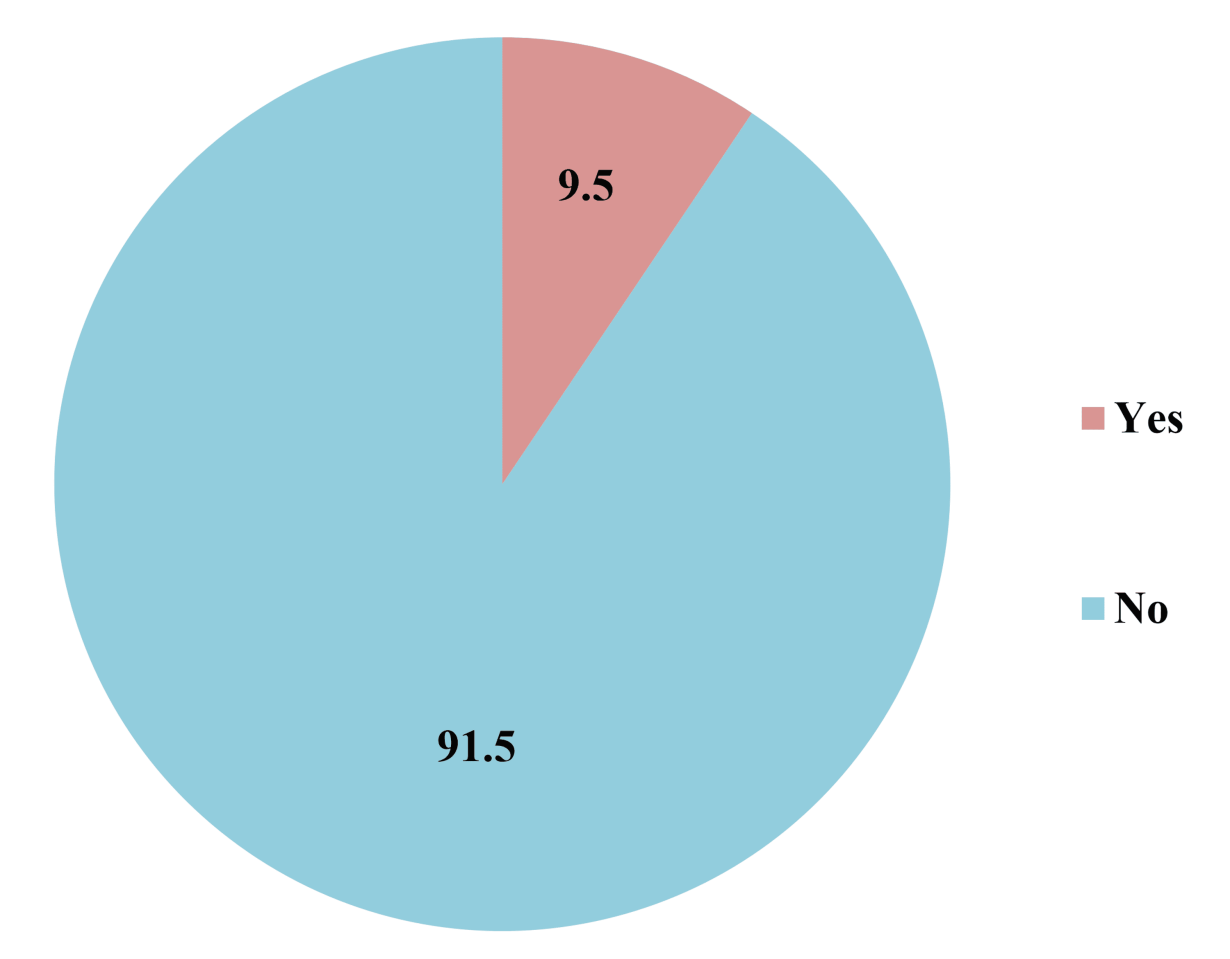
Proportion of non-alcoholic fatty liver disease (NAFLD) among study participants

Among the study participants, 17 (9.5%) were suffering from NAFLD (Figure 3).

**Figure 4:**
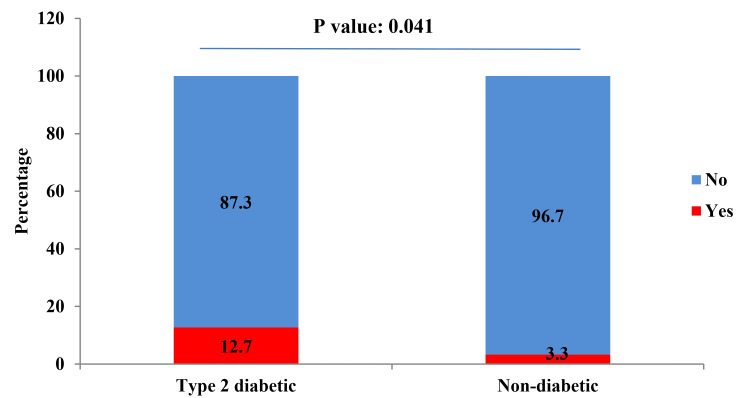
Proportion of non-alcoholic fatty liver disease (NAFLD) among type-2 diabetic and non-diabetic participants

Among the T2DM, 15 (12.7%) were suffering from NAFLD; and among non-DM patients, two (3.3%) were suffering from NAFLD. A significant association was observed between T2DM and NAFLD (p-value: 0.041) (Figure 4).

No significant association was found between socio-demographic characteristics and risk factors with NAFLD, with the exception of T2DM. The prevalence of T2DM among individuals in the NAFLD group was significantly higher than that in the non-NAFLD group. In the group of patients with NAFLD, five individuals (29.4%) reported a family history of liver disease, whereas among those without NAFLD, only 22 individuals (13.6%) had a similar family history. In patients with NAFLD, 12 (70.6%) reported a family history of diabetes, whereas among those without NAFLD, 77 (47.5%) had a family history of diabetes (Table 2).

**Table 2:** Association of socio-demographic and risk factors variables with non-alcoholic fatty liver disease (NAFLD) (N=179) SD- Standard deviation, SSC- Secondary school certificate, HSC- Higher Secondary school certificate ^a^ Chi-square test, ^b^ Fisher’s exact test was done Data were presented as frequency (percentage)

Variables		Non-Alcoholic Fatty Liver Disease		Total	Test value	P value
		Yes	No			
Age (years)	18-40	5 (29.4)	48 (29.6)	53 (29.6)	1.484	^a^ 0.476
	41-60	11 (64.7)	87 (53.7)	98 (54.7)		
	>60	1 (5.9)	27 (16.7)	28 (15.6)		
Gender	Male	7 (41.2)	65 (40.1)	72 (40.2)	0.007	^a^ 0.933
	Female	10 (58.8)	97 (59.9)	107 (59.8)		
Education	Below SSC	3 (17.6)	36 (22.2)	39 (21.8)	1.983	^a^ 0.739
	SSC	2 (11.8)	23 (14.2)	25 (14.0)		
	HSC	4 (23.5)	49 (30.2)	53 (29.6)		
	Honors	6 (35.3)	46 (28.4)	52 (29.1)		
	Masters and above	2 (11.8)	8 (4.9)	10 (5.6)		
Occupation	Employed	5 (29.4)	46 (28.4)	51 (28.5)	3.674	^a^ 0.597
	Unemployed	10 (58.8)	91 (56.2)	101 (56.4)		
	Businessman	1 (5.9)	3 (1.9)	4 (2.2)		
	Retired	0 (0.0)	13 (8.0)	13 (7.3)		
	others	0 (0.0)	5 (3.1)	5 (2.8)		
	Students	1 (5.9)	4 (2.5)	5 (2.8)		
Type-2 diabetes mellitus		15 (88.2)	103 (63.6)	118 (65.9)	4.163	^b^ 0.041
Hypertension		8 (47.1)	78 (48.1)	86 (48.0)	0.007	^a^ 0.932
High cholesterol level		11 (64.7)	109 (67.3)	120 (67.0)	0.046	^a^ 0.830
Family history of Diabetes mellitus		12 (70.6)	77 (47.5)	89 (49.7)	3.272	^a^ 0.070
Family history of liver disease		5 (29.4)	22 (13.6)	27 (15.1)	3.011	^b^ 0.144
History of cerebrovascular disease		0 (0.0)	3 (1.9)	3 (1.7)	0.320	^b^ >0.99
Hypothyroidism		1 (5.9)	24 (14.8)	25 (14.0)	1.022	^b^ 0.474
Obesity		12 (70.6)	118 (72.8)	130 (72.6)	0.039	^b^ 0.783
Adequate fruit and vegetable intake		15 (88.2)	152 (93.8)	167 (93.3)	0.769	^b^ 0.318
Routine physical exercise		4 (23.5)	40 (24.7)	44 (24.6)	0.011	^b^ >0.99

No significant difference in glycosylated hemoglobin (HbA1c), AST, ALT, low-density lipoprotein (LDL), high-density lipoprotein (HDL), total cholesterol (TC), and triglyceride (TG) was observed between participants with or without NAFLD. A significantly higher level of serum albumin level was observed in the NAFLD group (4.19±0.35 g/dl) than in participants without NAFLD (3.89 ±0.52 g/dL) (p-value: 0.022). A significantly higher level of random blood sugar level was observed in the NAFLD group (13.09±6.54 mmol/L) than in participants without NAFLD (10.93±5.73 mmol/L) (p-value: 0.038) (Table 3).

**Table 3:** Distribution of laboratory parameters among participants with or without non-alcoholic fatty liver disease (NAFLD) (N=179) ^a ^Mann-Whitney U test,^ b^ Student t-test was done Data were presented as frequency (percentage)

Variables		Non-Alcoholic Fatty Liver Disease		Test value	P value
		Yes	No		
Random blood sugar (RBS) (mmol/L)	Mean ±SD	13.09±6.54	10.93±5.73	-2.076	^a^ 0.038
	Median (range)	10.78 (8.0-35.66)	8.99 (4.10-36.50)		
Glycosylated hemoglobin (HbA1c) (%)	Mean ±SD	8.40±2.60	7.81 ±2.71	-1.272	^a^ 0.203
	Median (range)	7.3 (5.20-14.50)	7.15 (4.0-17.54)		
Total cholesterol (mg/dL)	Mean ±SD	169.85 ±66.02	164.61±64.03	-0.477	^a^ 0.633
	Median (range)	182.0 (54.36-296.00)	158.0 (18.50-399.0)		
Low-density lipoprotein (LDL) (mg/dL)	Mean ±SD	106.50±50.82	88.51±41.24	-1.480	^a^ 0.139
	Median (range)	106 (33.12-208)	77.0 (21.0-244)		
High-density lipoprotein (HDL) (mg/dL)	Mean ±SD	36.59±11.95	40.05 ±16.46	-0.536	^a^ 0.592
	Median (range)	39.0 (16.74-58.0)	40.0 (8.0-87.0)		
Triglyceride (TG) (mg/dL)	Mean ±SD	207.72±216.60	174.50 ±156.06	-0.334	^a^ 0.739
	Median (range)	178.0 (21.42-931.0)	144 (18.18-1400.0)		
Aspartate aminotransferase (AST) (U/L)	Mean ±SD	34.06±47.07	26.58 ±17.5	-0.458	^a^ 0.647
	Median (range)	22.00 (12.0-213.0)	22.0 (11.0-140.0)		
Alanine aminotransferase (ALT) (U/L)	Mean ±SD	46.31±45.58	33.23±21.31	-0.293	^a^ 0.770
	Median (range)	26.0 (13.0-191.0)	27.0 (14.0-177.0)		
Uric acid (mg/dL)	Mean ±SD	3.58±0.97	4.96±4.67	-1.656	^a^ 0.098
	Median (range)	3.70 (2.30-5.40)	4.24 (1.93-59.0)		
Platelet count (×10^9^/L)	Mean ±SD	256.29 ±74.13	286.04±100.76	-0.969	^a^ 0.332
	Median (range)	238 (155.0-389.0)	274.0(105.0-589.0)		
Serum albumin (g/dL)	Mean ±SD	4.19±0.35	3.89 ±0.52	-2.298	^a^ 0.022
	Median (range)	4.2 (3.61-4.90)	4.0 (2.41-6.10)		
Abdominal girth (cm)	Mean ±SD	100.06±10.09	99.10±13.17	-0.111	^a^ 0.945
	Median (range)	98.00 (84.0-117.50)	100.0 (54-133.0)		
Body mass index (kg/m^2^)	Mean ±SD	27.73±4.65	28.14 ± 5.26	-0.450	^b^ 0.732
	Median (range)	28.48 (19.46-36.70)	27.81 (15.37-45.91)		

A family history of liver disease increases the risk of suffering from NAFLD by 2.652 times (95% CI: 0.851-8.257) (p-value: 0.092). A family history of diabetes mellitus increases the risk of suffering from NAFLD by 2.649 times (95% CI: 0.893-7.863) (p-value: 0.079). T2DM increases the risk of suffering from NAFLD by 4.296 times (95% CI: 0.949-19.442) (p-value: 0.058) (Table 4).

**Table 4:** Univariate logistic regression of risk factors for non-alcoholic fatty liver diseases (NAFLD) Univariate logistic regression was done Data were presented as Odds ratio, 95% confidence interval

Risk factors	Odds ratio	95% confidence interval	P-value
Family history of liver disease	2.652	0.851-8.257	0.092
Family history of diabetes mellitus	2.649	0.893-7.863	0.079
Type 2 diabetes mellitus (T2DM)	4.296	0.949-19.442	0.058

T2DM and a family history of liver disease were found to significantly increase the risk of suffering from NAFLD by 5.247 times (95% CI: 1.081-25.468) (p-value: 0.040) and 4.202 times (95% CI: 1.249-14.135) (p-value: 0.020), respectively (Table 5). 

**Table 5:** Multivariate logistic regression of risk factors for non-alcoholic fatty liver disease (NAFLD) Multivariate logistic regression was done Data were presented as Odds ratio, 95% confidence interval

Risk factors	Odds ratio	95% confidence interval	P-value
Family history of liver disease	4.202	1.249-14.135	0.020
Family history of diabetes mellitus	1.712	0.538-5.445	0.363
Type 2 diabetes mellitus	5.247	1.081-25.468	0.040

**Figure 5:**
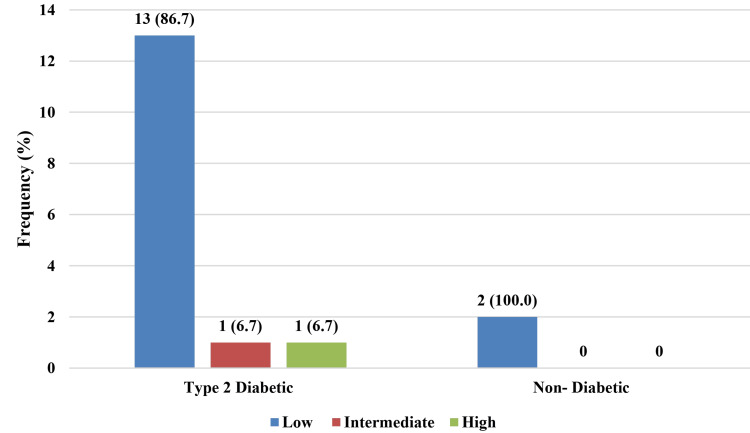
Distribution of the type-2 diabetic and non-diabetic participants suffering from non-alcoholic fatty liver disease according to level of risk fibrosis by FIB-4 score (n=17) FIB-4 - Fibrosis-4

About 13 (86.7%) of T2DM individuals with NAFLD were at low risk for fibrosis, whereas one (6.7%) was at high risk. All non-diabetic patients with NAFLD had a low risk of liver fibrosis (Figure 5).

## Discussion

NAFLD, currently termed MASLD, is an escalating public health concern and is considered the leading cause of chronic liver disease [23]. NAFLD frequently coexists with T2DM [18]. Studies at both national and international levels have highlighted diabetes as a potential risk factor for NAFLD. This study indicates that T2DM and a familial predisposition to liver disease significantly elevate the likelihood of NAFLD. Furthermore, NAFLD with T2DM is associated with heightened risks of fibrosis.

The study found a prevalence of NAFLD at 9.5%. An earlier ultrasonographic study of 2,782 adults in Bangladesh found that 34% of them had NAFLD [6]. Another study conducted by Rahman et al. among 1,305 community people reported that NAFLD was prevalent in 18.5% of the participants [8]. Differences in the survey method, study sites, participants (adult volunteers, rural or urban community people), and participants' metabolic status may have resulted in this variation in the prevalence. 

In Bangladesh, adults in midlife, specifically those aged 45 to 54 years, had the highest prevalence, with a subsequent decline in risk observed in persons over 55 years [6]. NAFLD is more prevalent in persons aged 40 to 60 years in India [24]. This study found no statistically significant relationship between age group and the prevalence of NAFLD; however, individuals aged 40-60 years showed a higher prevalence, while the proportion decreased in those over 60 years. This higher proportion of NAFLD patients in this age group may be the result of a sedentary lifestyle, dietary habits, an increased proportion of T2DM in this age group of participants, and metabolic changes associated with aging. These changes eventually lead to insulin resistance, hypertriglyceridemia, and obesity, which may ultimately result in NAFLD. This study indicates that both males and females were nearly equally impacted by hepatic fatty alterations, with a slight female predominance, consistent with the findings of Alam et al. [6]. Multiple hospital-based studies in Bangladesh have indicated a predominance of fatty liver among females [14,25,26]. In rural regions, women exhibited a nearly 10% higher susceptibility (1.27 times greater) to developing NAFLD compared to men [6]. In rural regions, women typically remain at home due to social conservatism, resulting in a sedentary lifestyle. This may contribute to the female predominance of NAFLD in rural regions [26]. 

Being diabetic, suffering from Raised blood pressure, dyslipidemia, metabolic syndrome, and central and generalized obesity are all known to be related to NAFLD in the South Asian region [27]. Insulin resistance, hyperinsulinemia, and chronic hyperglycemia associated with T2DM promote lipolysis in adipose tissue, enhance de novo lipogenesis, impede free fatty acid uptake by the liver, and induce oxidative stress and inflammation, leading to hepatocellular injury and ultimately contributing to NAFLD [28]. Approximately two-thirds of the individuals involved in this study had T2DM. About 15 (12.7%) of the T2DM patients were suffering from NAFLD, which was higher than the proportion of the overall participants with NAFLD. This much higher proportion of NAFLD in diabetic individuals than in the general population was also observed by Alam et al. [5]. The presence of T2DM was associated with a 5.247-fold increase in the risk of NAFLD, with a 95% confidence interval of 1.081 to 25.468. According to Alam et al., people with diabetes had an increased chance of developing NAFLD (adjusted odds ratio: 2.71, 95% CI: 1.85-3.97) [6]. According to the findings of Rahman et al., diabetes elevates the risk of NAFLD by a factor of 2.21, with a confidence interval ranging from 1.43 to 3.41 [8]. This study revealed that nearly 7% of diabetic individuals with NAFLD were at higher risk of fibrosis, while all non-diabetic patients with NAFLD exhibited a low risk of liver fibrosis. Variations in the method used to determine NAFLD, as well as variations in study location and population, may account for the observed discrepancy in the probabilities of increasing risk. In addition, a family history of liver disease was identified as a factor that increases the risk of developing NAFLD by 4.202 times (95% CI: 1.249-14.135) (p-value: 0.020), corroborating the findings reported by Alam et al. [6].

A familial predilection to DM has been shown to double the likelihood of NAFLD, independent of confounding factors [29]. Although individuals with NAFLD showed a significantly higher rate of family history of diabetes, this study found no appreciable relationship. No notable association between dyslipidemia and NAFLD was detected. The risk of developing NAFLD was 4.51 times higher in overweight people compared to normal-weight people, according to research by Alam et al. [6]. Fatty liver is prevalent in individuals with increased BMI. Nonetheless, this study did not reveal any such link. Emerging, though limited, evidence suggests that NAFLD could impact individuals who are of normal weight or slender, especially within the Asian population. A recent systematic review and meta-analysis revealed that the global prevalence of non-obese or lean NAFLD is 12.1% in the general population and 8.6% in community-based studies. Non-obese NAFLD was observed in 10% of the population in Southern Asia [30]. Considering the above-mentioned facts, patients with T2DM and with a positive family history of liver disease should be on regular follow-up for timely diagnosis of NAFLD and patients with NAFLD should assess the fibrosis risk to ensure proper evidence-based management.

Limitation

This research employed a cross-sectional approach, which renders the establishment of a direct causal relationship. It was an urban hospital-based study, so it might not represent the community scenario and known factors like insulin resistance, and metabolic syndrome were not evaluated.

## Conclusions

NAFLD was prevalent in middle-aged and women. Approximately one in 10 participants were affected by this condition. Non-alcoholic fatty liver disease was prevalent among type 2 diabetic patients, who also exhibit a higher risk of liver fibrosis. T2DM and a familial history of liver disease were identified as independent predictors of NAFLD. To promote optimal hepatic health and efficient use of limited healthcare resources, patients at risk should undergo regular follow-up, and those with NAFLD must have their fibrosis status assessed to evaluate the risk of cirrhosis or hepatic carcinoma. Furthermore, there is a necessity for additional multicenter studies with larger sample sizes, along with public health interventions to effectively manage and prevent NAFLD and its adverse health consequences.
